# Use of Urodynamics by Gynecologists and Urologists in Brazil

**DOI:** 10.1055/s-0042-1744460

**Published:** 2022-08-08

**Authors:** Mucio Barata Diniz, Marina Franklin Ribeiro, Luísa Aguiar Monteiro Dias, Marilene Vale de Castro Monteiro

**Affiliations:** 1 Department of Gynecology and Obstetrics of Federal University of Minas Gerais, Belo Horizonte, Brazil; 2 Gynecology Unit of Vila da Serra Hospital, Belo Horizonte, Brazil

**Keywords:** urodynamics, female urinary incontinence, overactive bladder, urodinâmica, incontinência urinária feminina, bexiga hiperativa

## Abstract

**Objective** Urodynamic studies (UDSs) are a set of tests that assess the storage and emptying of urine, and they are widely used by gynecologists and urologists in the management of urinary incontinence (UI), despite the discussion about its indications. The objectives of the present study were to verify whether UDSs are routinely used in the conservative and surgical approaches to female UI, their other clinical indications, and to compare the responses of Brazilian gynecologists and urologists.

**Methods** The present is an opinion survey applied from August 2020 to January 2021 through a semistructured questionnaire about the clinical practice sent by e-mail to all participants. The responses were compared through statistical analyses.

**Results** Of the 329 participants, 238 were gynecologists (72.3%) and 91, urologists (27.7%). Most gynecologists (73.5%) and urologists (86.6%) do not request UDSs before the conservative treatment of UI; but UDSs are indicated in the preoperative period of anti-incontinence surgeries. Most participants request UDSs in the initial approach to overactive bladder (gynecologists: 88.2%; urologists: 96.7%), and the urologist has greater chance to request this study (odds ratio [OR] = 3.9). For most participants, it is necessary to request uroculture before the UDSs.

**Conclusion** Most Brazilian gynecologists and urologists who participated in the present study do not request UDSs before the conservative treatment of UI, according to national and internacional guidelines, and often request it before the surgical treatment for female UI. The indication of this exam in the initial approach of idiopathic overactive bladder should be reviewed by the participants.

## Resumo

**Objetivo** O estudo urodinâmico (EU) é um conjunto de exames que avalia o armazenamento e esvaziamento da urina, e é amplamente utilizado por ginecologistas e urologistas no manejo da incontinência urinária (IU), apesar das discussões sobre suas indicações. O objetivo deste estudo foi verificar se a urodinâmica é rotineiramente utilizada nas abordagens conservadora e cirúrgica da IU feminina, além de outras de suas indicações clínicas, e comparar as respostas de ginecologistas e urologistas brasileiros.

**Métodos** Trata-se de uma pesquisa de opinião, realizada entre agosto de 2020 e janeiro de 2021, por meio de questionário semiestruturado composto por perguntas sobre a prática clínica enviado por e-mail a todos os participantes. As respostas foram comparadas mediante análises estatísticas.

**Resultados** Dos 329 participantes, 238 eram ginecologistas (72,3%) e 91, urologistas (27,7%). A maioria dos ginecologistas (73,5%) e urologistas (86,6%) não solicita EU antes do tratamento conservador da IU; mas o EU é indicado no pré-operatório de cirurgias para IU. A maioria dos participantes solicita EU na abordagem inicial da bexiga hiperativa (ginecologistas: 88,2%; urologistas: 96,7%), e os urologistas têm maior chance de solicitar esse exame (razão de chances [RC] = 3,9). Para a maioria dos entrevistados, é necessário solicitar urocultura junto com o EU.

**Conclusão** A maioria dos ginecologistas e urologistas brasileiros que participaram deste estudo não solicita EU antes do tratamento conservador da IU, de acordo com as principais diretrizes nacionais e internacionais, e muitas vezes o solicita antes do tratamento cirúrgico da IU feminina. A indicação desse exame na abordagem inicial da bexiga hiperativa idiopática deve ser revista pelos participantes.

## Introduction

Urodynamic studies (UDSs) are a set of tests that evaluate the storage and emptying of urine, and they are widely used by gynecologists and urologists in the management of urinary incontinence (UI) and to assess the function of the lower urinary tract. The objective of UDSs is to reproduce the patient's symptoms and make the pathophysiological correlation, identifying the factors that contribute to urinary tract dysfunction. 1 2 The International Continence Society (ICS) recommends performing at least three stages of this exam, which are flowmetry, cystometry, and the pressure-flow study. 3 4

The approach to female UI is divided into initial and specialized. 5 The initial approach should include: anamnesis, physical examination with the stress test, urinalysis, urinary diary, and assessment of residual urinary volume. 6 Recent guidelines 6 7 8 9 suggest that, when the conservative treatment fails or when UI is defined as complicated, additional tests are needed, with UDSs are the main one. Patients classified as complicated UI are those with urine leakage associated with prolapse or urgency, patients with bladder symptoms emptying, those undergoing radical pelvic surgery or radiotherapy, those who have recurrences and patients in whom the initial approach did not define the clinical diagnosis. 5 9 10 Despite their importance as functional tests, the role of UDSs in evaluating female patients with UI remains under debate regarding the situations in which they should be indicated. 11

In order to know the indications for UDSs made by gynecologists and urologists in Brazil, where there is no filed of expertise in urogynecology, we performed a survey. The objectives of the present study were to verify whether UDSs are routinely used in the conservative and surgical approaches to female UI, in what other clinical situations they are requested by the participants, and to compareing the responses of gynecologists and urologists.

## Methods

The present is an opinion survey aimed at Brazilian gynecologists and urologists and applied through a semistructured questionnaire. The study was approved by the Ethics in Research Committee of Universidade Federal de Minas Gerais (under CAAE: 34191120.5.0000.5149), and was carried out between August 2020 and January 2021. The questionnaire was sent by email, by Federação Brasileira das Associações de Ginecologia e Obstetrícia (Brazilian Federation of Gynecology and Obstetrics Associations, Febrasgo, in Portuguese) and Sociedade Brasileira de Urologia (Brazilian Society of Urology, SBU, in Portuguese), to 30 thousand gynecologists and urologists, and before answering, those who were willing to participate marked the consent form and were not identified after filling out the questionnarie.

The questionnaire consisted of questions about the clinic practice and requests for UDSs in approaching to female IU, and was developed by two specialists in gynecology and urology.

The main objective was to verify the percentage of participants who routinely requested UDSs before starting the conservative or surgical treatments of female UI. The other objectives were: to confirm whether UDSs are requested before the surgical treatment of female UI; to assess the main clinical conditions for which the participants request UDSs; to assess the availability of UDSs in the participants' location; to identify whether the surgical treatment for UI was based on the pressure of urine leakage; and to assess whether there was a difference in UDS indications between gynecologists and urologists. The sample calculation was not performed because it is an opinion poll.

The numerical variables are expressed in terms of their values of central tendency and variability, considering the nature of their distribution. The categorical variables are expressed in terms of absolute and relative frequencies. For the descriptive analysis of th variables with normal distribution, the results were expressed as means ± standard deviations. To compare the responses of gynecologists and urologists, the Student *t* -test was used, after the performance of the Levene test to verify the homogeneity of variances by group. For the categorical variables, the Pearson Chi-squared test (χ ^2^ ) and the Fisher exact test were also used. In cases of significant association between two variables of interest, the odds ratio (OR) was evaluated. In all statistical calculations, the confidence level was set a 0.95. The statistical analysis was performed using the Statistical Package for the Social Sciences (IBM SPSS Statistics for Windows, IBM Corp., Armonk, NY, United States)software, version 21.0.

## Results

Of 30 thousand questionnaries sent, only 329 (1.1%) were filled out. Out of those 329 participants, 238 (72.3%) were gynecologists and 91 (27.7%), urologists. Regarding the years of experience in the specialty, the average was of 21.2 years among the gynecologists, and most were female (60.9%), and 17.5 years among the urologists (93.4% of them male), with a statistically significant difference ( *p* = 0.023 for of the years of professional experience, and *p* = 0.001 for gender). There was no statistically significant difference regarding professional qualification (postgraduate courses and specialization). As for the location where they work, most gynecologists worked in the capital city of their states (55.5%), but only 39.6% of urologists worked in the capital city ( *p* = 0.023) ( Table 1 ).

**Table 1:** Characteristics of study participants

	Gynecologist N (%)	Urologist N (%)	*p* -value ^a^
Number of participants	238 (72,3%)	91 (27,7%)	
Female	145 (60,9%)	6 (6,6%)	**0,001**
Male	93 (39,1%)	85 (93,4%)	
Average years of professional experience	21,2 years	17,5 years	0,023**
Postgraduate or specialization	63 (26,5%)	19 (20,9%)	0,294
Practice in state's capital Practice in countryside	132 (55,5%) 99 (41,6%)	36 (39,6%) 50 (54,9%)	0,023*
The UDS is available in your region	235 (98,7%)	91 (100%)	0,564*

Notes: ^a^ Pearson Chi-squared test; *Fisher exact test; **Student t-test for independent samples.

Urodynamic studies were available to the vast majority of participants (98.7% of gynecologists and 100% of urologists); 73% of gynecologists and 88% of urologists indicate UDSs in the preoperative period of anti-incontinence surgeries, and there was no statistical difference between the two groups; 53.4% of gynecologists and 62.6% of urologists do not indicate UDSs in the preoperative period of surgeries for genital prolapse, with no statistical difference between groups; and most gynecologists (73.5%) and urologists (86.6%) do not request UDSs before starting the conservative treatment of UI ( Table 2 ).

**Table 2:** Indications of the urodynamic study

Indications	Gynecologist N= 238	Urologist N= 91	*p* -value*
Do you recommend the urodynamic “in the preoperative period of anti-incontinence surgeries”?			
yes no	175 (73,5%) 63 (26,5%)	75 (82,4%) 16 (17,6%)	0,091
Why do you request the urodynamic before anti-incontinence surgery?			
For Sling systhem Release Becausee it is part of the protocol For decision shared with the patient For legal certainty	84 (35,3%) 25 (10,5%) 97 (40,8%) 64 (26,9%)	38 (41,8%) 8 (8,8%) 57 (62,2%) 48 (52,7%)	
Do you recommend the urodynamic in “patients with genital prolapse with indication for surgical treatment”?			
yes no	111(46,6%) 127 (53,4%)	34 (37,4%) 57 (62,6%)	0,130
Do you recommend the urodynamic in “patients with mixed incontinence”?			
Yes Not	129 (54,2%) 109 (45,8%)	48 (52,7%) 43 (47,3%)	0,813
Do you indicate the urodynamics “in the initial approach of idiopathic OAB”?			
Yes Not	210 (88,2%) 28 (11,85%)	88 (96,7%) 3 (3,3%)	**< 0,001** **OR= 3,9**
Do you request the urodynamic “before indicating conservative treatment”?
Yes Not	63 (26,5%) 175 (73,5%)	12 (13,2%) 79 (86,6%)	**0,010** **OR= 2,4**
The result of the urodynamic interferes with the type of anti-incontinence surgery”?			
Yes Sometimes Not Does not indicate surgery	115 (48,3%) 76 (32%) 31(13%) 16 (6,7%)	22 (24,2%) 36 (39,5%) 33 (36,3%) –	**0,001**

Abbreviaitons: OAB, overactive bladder; OR, odds ratio.

Note: *Pearson Chi-squared test.

When asked about UDSs in cases of mixed incontinence, 54.2% of gynecologists and 52.7% of urologists indicated them. There was a statistical difference regarding the indication of UDSs in the approach to idiopathic overactive bladder (OAB), as urologists indicated this less frequently than gynecologists: 3.3% and 11.8% respectively ( Table 2 ). Most urologists perform UDSs (71.4%) as opposed to gynecologists (27.7%), which was statistically significant ( *p* = 0.001). Among the participants who perform UDSs, most use two urethral catheters, use a device made in Brazil, and perform the three main exams that are part of UDSs (uroflowmetry, cystometry, and pressure-flow study). When we evaluated the protocol for the performance of UDSs, we only observed a difference regarding the use of prophylactic antibiotics, which was greater among urologists. The main piece of data from the UDSs to indicate anti-continence surgery was the pressure of urinary loss, both for gynecologists and urologists ( Table 3 ).

**Table 3:** Routine of the urodynamic study

	Gynecologist N= 238	Urologist N = 91	*p* -value*
Do you perform urodynamic study? Yes Not	66 (27,7%) 172 (72,3%)	65 (71,4%) 26 (28,6%)	**0,001**
Which urinary catheter do you use 2 relief catheters double lumen catheter	44 (78,6%) 12 (21,4%)	49 (75,4%) 16 (24,6%)	
Your urodynamic device is: National Imported	57 (83,3%) 9 (13,7%)	63 (96,9%) 2 (3,1%)	
Inform which item below is part of your urodynamic protocol: Previous Uroculture Anamnesis Prophylactic antibiotic validated urinary incontinence questionnaires	58 (87,9%) 60 (90,9%) 24 (36,4%) 39 (59,1%)	58 (89,2%) 62 (95,4%) 37 (56,9%) 37 (56,9%)	
What urodynamic tests do you routinely perform in the workup of female UI? Uroflowmetry Cystometry Measurement of residual volume after uroflowmetry Urethral pressure profile	57 (100%) 57 (100%) 46 (80,7%) 9 (15,8%) 52 (91,2%)	56 (86,2%) 61 (93,8% 54 (83,1%) 2 (7,7%) 64 (98,5%)	
What is the main UDS data to indicate anti-incontinence surgery? loss pressure	174 (73,1%)	68 (74,7%)	
What is the average cost of the urodynamic study?	R$ 397,00	R$ 503,00	

Note: _Pearson Chi-squared test.

## Discussion

Most Brazilian gynecologists and urologists participating in the present study do not request UDSs before starting the conservative treatment of UI. Most Brazilian gynecologists and urologists participating in the present study do not request UDSs before starting the conservative treatment of UI; and this clinical approach is in accordance with the main national and international protocols and guidelines that show there is no evidence that performing UDSs before the conservative treatment will result in lower rates of subsequent UI. 12 13 However, gynecologists indicate UDSs more frequently in this situation than urologists (OR = 2.4). There is a consensus that these tests should not be indicated in the initial assessment of uncomplicated female UI. 6 7 8 11

On the other hand, most participants request UDSs before the surgical treatment of female UI, with no statistical difference between gynecologists and urologists (73% and 88% respectively). Although the indications for UDSs are controversial, their performance can be waivered preoperatively in cases of uncomplicated UI, as shown in the study by Nager et al. (2012), 14 who did not observe significant differences in the surgical outcomes of patients who did or did not undergo the exams. Routine UDSs in the preoperative period of uncomplicated stress UI (SUI) is not recommended by Febrasgo, the European Association of Urology (EAU), England's National Institute of Excellence in Health and Care (NICE), or the American College of Gynecology and Obstetrics (ACOG). 7 10 13 15 Other authors point out that there are situations in which UDSs provide additional information to the clinical assessment, even in cases of uncomplicated SUI, and that these exams should be requested mainly in cases of suspected bladder-emptying dysfunction. 16 17 18 When listing the main reasons to request UDSs preoperatively, the participants responded: in order to obtain authorization to use the synthetic sling (both in the private and public health care systems), because it is part of their institution's protocol, in orderto share decisions with the patient, and due to legal concerns. The other clinical indications for UDSs evaluated were genital prolapse, OAB, and mixed incontinence.

Regarding genital prolapse, 53% of gynecologists and 62% of urologists request UDSs preoperatively, with no statistical difference between groups. It was not possible to identify the main reason for this request, but it may be related to the investigation of occult UI and for the indication of anti-incontinence surgery in the same surgical act. 19 20 21 In these cases, UDSs would be indicated for patients complaining of urine loss concomitant with prolapse or for the diagnosis of occult UI. 17 19

Most gynecologists and urologists indicate UDSs in the initial approach to OAB (88.2% and 96.7% respectively), and there was a statistically significant difference between the groups ( *p* = 0.001). There is a greater chance that urologists will request UDSs in this situation compared to gynecologists (OR = 3.9). There is no indication for UDSs in the initial approach to idiopathic OAB. 6 7 16 21 This finding suggests the need to review the care protocols of the participants for idiopathic OAB.

Although most gynecologists (54.2%) and urologists (52.7%) indicate UDSs in the management of mixed UI, we observed that more than 40% of the participants do not request it for this clinical condition. The fact that there is no question about mixed urinary incontinence in the questionnaire may have contributed to this finding, and there may be a correlation with the fact that most participants request UDSs for OAB. 11

Overall, UDSs are available to most participants. Comparing the both groups, there are more women in gynecology (60.9%) than in urology (6.6%). Most participants were gynecologists. Despite the voluntary random sample, gynecologists probably treat more women with UI than urologists; therefore, they had greater participation in the questionnaire. However, urologists (71%) perform more UDSs than gynecologists (27%).

Ideally, cystometry and the pressure-flow study should be performed with a double-lumen catheter, but a minority of participants use this catheter (21.4% of gynecologists and 24.6% of urologists), probably because t is 15 times more expensive than the two urethral catheters. 3 The recording of leak pressure during cystometry was the main piece of data in the UDSs to indicate anti-incontinence surgery by most participants (73.1% of gynecologists and 74.7% of urologists).

Urinary tract infection is the most common complication after UDSs, estimated in 8.4% of cases, and the main risk factors are advanced age, diabetes mellitus, genital prolapse, previous anti-incontinence surgery, and recent urinary tract infection. 20 21 The ACOG does not recommend antibiotic prophylaxis for UDSs, and a recent systematic review 21 concluded that there are insufficient studies to recommend its routine use. However, Cameron et al. 22 recommend a single oral dose of antibiotics before UDSs for women with neurogenic dysfunction, high postvoiding residual volume, asymptomatic bacteriuria, immunosuppression, age over 70 years, and those using an indwelling urinary catheter or intermittent catheterization. 22 23 The use of prophylactic antibiotics before UDSs was indicated by 36.4% of gynecologists and 56.9% of urologists in the present study.

The main limitations of the present study were not classifying complicated and uncomplicated UI for each question in the questionnaire, and not correlating the request for UDSs in cases of genital prolapse with the investigation of occult UI. Another important limitation of our study was the participation of less than 10% of gynecologists and urologists registered in Brazil. These participants are probably more interested in female UI, especially gynecologists, who accounted for the majority of the sample. This is a limiting factor to extend our conclusion to all gynecologists and urologists in Brazil. The relevance of the present study was the characterization of the main indications for UDSs in this sample of Brazilian gynecologists and urologists. 24 25 26 27

## Conclusion

Most Brazilian gynecologists and urologists participating in the present study do not request UDSs before the conservative treatment of UI, according to national and internacional guidelines, and often request these exams before the surgical treatment of female UI. The indication for these exams in the initial approach of idiopathic OAB should be reviewed by the participants.
